# Karyotype and genome size comparative analyses among six species of the oilseed-bearing genus *Jatropha* (Euphorbiaceae)

**DOI:** 10.1590/1678-4685-GMB-2017-0120

**Published:** 2018-05-14

**Authors:** Anne C.T.A. Marinho, Santelmo Vasconcelos, Emanuelle V. Vasconcelos, Daniela A. Marques, Ana Maria Benko-Iseppon, Ana Christina Brasileiro-Vidal

**Affiliations:** 1 Universidade Federal de Pernambuco Universidade Federal de Pernambuco Department of Genetics RecifePE Brazil Department of Genetics, Universidade Federal de Pernambuco, Recife, PE, Brazil; 2 Instituto Tecnológico Vale Instituto Tecnológico Vale BelémPA Brazil Instituto Tecnológico Vale, Belém, PA, Brazil; 3 Instituto Agronômico Instituto Agronômico Centro de P&D de Recursos Genéticos Vegetais CampinasSP Brazil Centro de P&D de Recursos Genéticos Vegetais, Instituto Agronômico, Campinas, SP, Brazil

**Keywords:** Cytotaxonomy, DNA C-value, heterochromatin, physic nut, rDNA

## Abstract

*Jatropha* is an important genus of Euphorbiaceae, with species largely used for various purposes, including the manufacturing of soaps and pharmaceutical products and applications in the bioenergetic industry. Although there have been several studies focusing *J. curcas* in various aspects, the karyotype features of *Jatropha* species are poorly known. Therefore, we analyzed six *Jatropha* species through fluorochrome staining (CMA/DAPI), fluorescent *in situ* hybridization (FISH) with 5S and 45S rDNA probes and genome size estimation by flow cytometry. Our results revealed several chromosome markers by both CMA/DAPI and FISH for the analyzed species. Five *Jatropha* species (*J. curcas*, *J. gossypiifolia*, *J. integerrima*, *J. multifida* and *J. podagrica*) showed four CMA-positive (CMA^+^) bands associated with the 5S and 45S rDNA sites (one and two pairs, respectively). However, *J. mollissima* displayed six CMA^+^/DAPI^-^ bands co-localized with both 5S and 45S rDNA, which showed a FISH superposition. A gradual variation in the genome sizes was observed (2C = 0.64 to 0.86 pg), although an association between evidenced heterochromatin and genome sizes was not found among species. Except for the unique banding pattern of *J. mollissima* and the pericentromeric heterochromatin of *J. curcas* and *J. podagrica*, our data evidenced relatively conserved karyotypes.

Euphorbiaceae is one of the most complex and diverse angiosperm families, presenting a worldwide distribution, mainly in the Americas and in Africa. The group has approximately 8,000 species, including several genera with remarkable economic importance, such as *Hevea* (the rubber tree genus), *Manihot* (cassavas), *Ricinus* (castor) and *Jatropha* (Webster, 1987; Souza and Lorenzi, 2008). The genus *Jatropha* is composed of approximately 200 species (Webster, 1994; The Plant List, 2013), which present a vast biotechnological potential due to outstanding characteristics, such as drought tolerance, secondary metabolites with medicinal properties and high seed oil content and quality. The species *J. gossypiifolia* and *J. podagrica*, for instance, have an extensive use as ornamental plants, while *J. ribifolia* have been used as raw material for soaps and detergents. Both the oil and latex have been drawing attention by the pharmaceutical industry, due to their active principles that can be used in the production of antiseptics, antifungals, healing drugs, laxatives, among other products (e.g., Anani *et al.*, 2016; Shahinuzzaman *et al.*, 2016; Sharma *et al.*, 2016). On the other hand, fruits and seeds, mainly from *J. curcas*, present high oil content, also serving as a strategic crop to be used as an alternative raw material for the production of biofuels (Openshaw, 2000; Montes and Melchinger, 2016).

The domestication process and the enhancement of traits of interest of *J. curcas* is still in the beginning (Yue *et al.*, 2013; Montes and Melchinger, 2016), although there has been a constant increase in the knowledge on the genetic variability of the species in the last decade (see, for instance, Guo *et al.*, 2016), being improved considerably since the publication of the genome by Sato *et al.* (2011). Nevertheless, the vast majority of the species of *Jatropha* is poorly known, lacking even characterization studies aiming interspecific genetic similarities and karyotype information (Marques and Ferrari, 2008; Ovando-Medina *et al.*, 2011, Marques *et al.*, 2013).

In the general sense, *Jatropha* is supposed to present a high karyotypic stability, with the diploid number 2*n* = 22 being reported for almost all the 31 analyzed species so far (see Rice *et al.*, 2015), including *J. curcas*, *J. gossypiifolia*, *J. integerrima*, *J. mollissima*, *J. multifida* and *J. podagrica*, although *J. cuneata* Wiggins & Rollins and *J. dioica* Sessé were reported as tetraploids (2*n* = 44) (Miller and Webster, 1966; Dehgan and Webster, 1979; Sasikala and Paramathma, 2010). Also, both the chromosome morphologies and sizes have been reported as highly stable within the genus, with a predominance of small metacentric and submetacentric chromosomes (Deghan and Webster, 1979; Carvalho *et al.*, 2008).

Basically, *J. curcas* is the only species with more refined analyses published, other than just chromosome counts, although there is an available genome size estimation for *J. podagrica* (2C = 0.60 ± 0.05 pg; Vesely *et al.*, 2012). Carvalho *et al.* (2008), for instance, presented a detailed karyotype analysis for *J. curcas* through standard staining and flow cytometry procedures, observing both small DNA content (2C = 0.85 ± 0.01 pg) and small chromosomes (ranging between 1.24-1.71 μm). Some authors have been assessing the physical distribution of large repetitive DNA clusters, such as rDNAs 5S and 45S, as well as different *copia*-type retrotransposons and subtelomeric repetitions in the *J. curcas* karyotype, observing several chromosome markers for the species (Witkowska *et al.*, 2009; Kikuchi *et al.*, 2010; Alipour *et al.*, 2013; Gong *et al.*, 2013).

The economic importance of *Jatropha* species is noteworthy, and there is an evident need for more information regarding the genetic differentiation within the genus. Therefore, this work aimed to describe cytogenetic markers by means of CMA/DAPI banding and FISH with 5S and 45S rDNA probes, besides providing genome size estimates, analyzing six species (Supplementary Table S1) largely used by several industry sectors, in order to contribute to a better understanding of the karyotype evolution of the genus.

Six *Jatropha* species were analyzed: *J. curcas* L., *J. gossypiifolia* L., *J. integerrima* Jacq., *J. multifida* L., *J. mollissima* (Pohl) Baill. and *J. podagrica* Hook (Figure 1). Root tips were collected either from germinated seeds or seedlings, pre-treated with 2 mM 8-hydroxyquinolein (8-HQ) for 4.5 h at 18 °C. The material was fixed in methanol:acetic acid (3:1, v/v) for at least 4 h and then stored at -20 °C. The preparation of slides followed the protocol described by Carvalho and Saraiva (1993), with modifications introduced by Vasconcelos *et al.* (2010).

**Figure 1:**
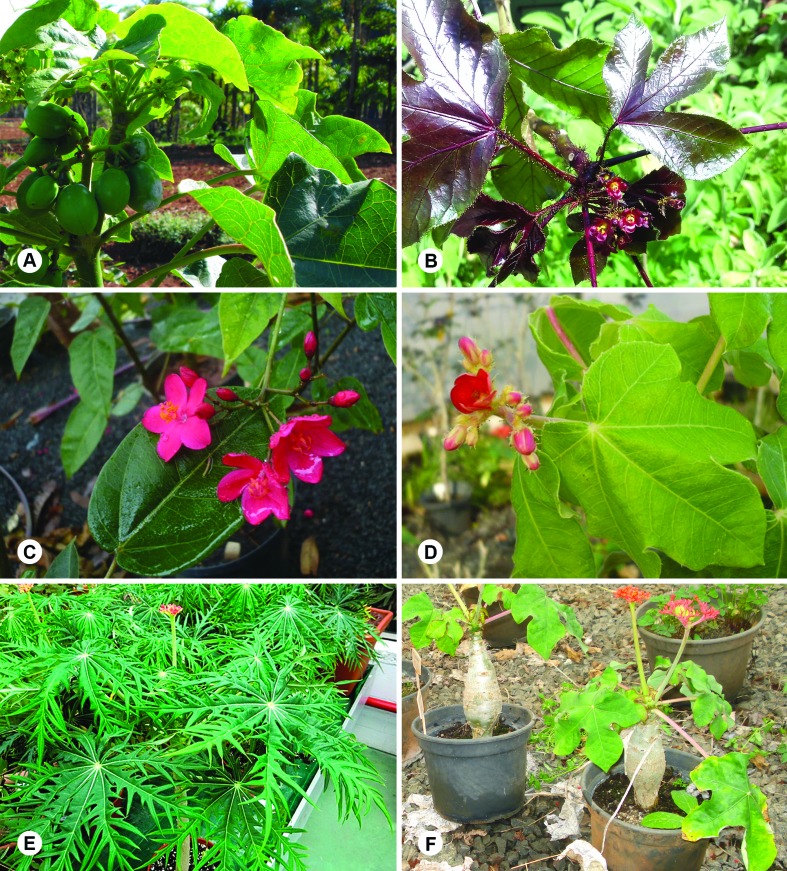
Representatives of the six analyzed *Jatropha* species: (A) *Jatropha curcas*; (B) *J. gossypiifolia*; (C) *J. integerrima*; (D) *J. mollissima*; (E) *J. multifida*; and (F) *J. podagrica*.

After preparation, the slides were stored for three days at room temperature (~25 °C) and then stained with 0.5 mg/mL CMA for 1 h and 2 μg/mL DAPI for 30 min, mounted in McIlvaine’s buffer (pH 7.0):glycerol (1:1, v/v) and stored for another three days (Schweizer and Ambros, 1994). After image capture, slides were destained in ethanol:acetic acid (3:1, v/v) for 30 min at room temperature, followed by immersion in ethanol for 1 h and storage at -20 °C.

The following probes were used in the FISH procedures: (1) R2, a 6.5 kb fragment containing the 18S-5.8S-25S rDNA repeat unit from *Arabidopsis thaliana* (L.) Heynh. (Wanzenböck *et al.*, 1997), and (2) D2, a 400 bp containing two 5S rDNA repeat units from *Lotus corniculatus* L. [as *L. japonicus* (Regel) K.Larsen] (Pedrosa *et al.*, 2002), which were labeled by nick translation with digoxigenin-11-dUTP (Roche Diagnostics) and biotin-11-dUTP (Sigma), respectively. The FISH pre-treatment and post-hybridization washes followed Pedrosa *et al.* (2002), in which the stringency wash (77%) was performed with 0.1X SSC at 42 °C. Chromosome and probe denaturation and detection were performed according to Heslop-Harrison *et al.* (1991). Ten microliters of the hybridization mixture, which contained 50% formamide (v/v), 2X SSC, 10% dextran sulfate (w/v) and 2.5-5 ng/μL of the probe, were added to each slide, being hybridized at 37 °C for at least 18 h. Detection of the digoxigenin-labelled probes was carried out using sheep anti-digoxigenin-FITC (Roche Diagnostics), and the signal was amplified with donkey anti-sheep-FITC (Sigma), in 1% (w/v) BSA. Biotin-labelled probes were detected with mouse anti-biotin (Dako), and the signal was visualized with rabbit anti-mouse TRITC conjugate (Dako), in 1% (w/v) BSA. Preparations were counterstained and mounted with 2 μg/mL DAPI in Vectashield (Vector) (1:1; v/v).

Images of the cells were captured on a Leica DMLB microscope with a Leica DFC 340FX camera, using the software Leica CW4000, with optimization for contrast and brightness using Adobe Photoshop CC (Adobe Systems Incorporated) software.

The DNA 2C-values were measured by using approximately 20-30 mg of fresh leaves of the six species, each with an internal reference standard (*Solanum lycopersicum* cv. Stupicke polni tyckove, 2C = 1.96 pg), being chopped in 1 mL of WPB buffer (Loureiro *et al.*, 2007), following the procedures described by Dolezel *et al.* (1989). The nuclei suspension was filtered through a 30 μm nylon mesh and then stained with 30 μL of 1% propidium iodide (w/v). Three individuals per species were analyzed and three replicates per individual, with at least 10,000 nuclei per sample, using the Partec CyFlow Space flow cytometer. Each histogram obtained from the relative fluorescence of the nuclei of the samples and the internal reference was analyzed in the software Partec FloMax 2.4. Afterward, the mean DNA 2C-values were calculated after discarding both the smallest and the largest readings obtained for each species.

All analyzed karyotypes presented the diploid number 2*n* = 22, with chromosomes predominantly metacentric and submetacentric (Table 1; Figures 2 and 3), as well as semi-reticulated interphase nucleus, confirming previous counts for all six species (Perry, 1943; Miller and Webster, 1962; Miller and Webster, 1966). Additionally, at least two satellited chromosomes were visualized for all six species.

**Table 1:** Karyotype characterization of the six analyzed *Jatropha* species, showing diploid chromosome numbers (2*n*); distribution pattern of CMA+/DAPI bands and 5S and 45S rDNA sites; genome size estimation (pg) and mean CV (%) per species.

Species	Accessions and provenances	2*n*	CMA^+^/DAPI^-^	45S rDNA	5S rDNA	2C (pg)	CV (%)
*J. curcas* L.^1^	LGBV-S2860; Embrapa Algodão, Patos, PB, Brazil	22	4 T^2^, P^3^	4 T	2 ST^4^	0.86 ± 0.02	3.95
*J. gossypiifolia* L.	LGBV-S3313; Engenho Ubu, BR 101 Norte, Km 24, Goiana, PE, Brazil	22	4 T, P	4 T	2 ST	0.64 ± 0.02	4.14
*J. integerrima* Jacq.	IAC-VI23; Instituto Agronômico (IAC), Campinas, SP, Brazil	22	4 T	4 T	2 ST	0.85 ± 0.03	4.27
*J. mollissima* (Pohl) Baill.^1^	LGBV-S3314; Parque Nacional do Catimbau, Buíque, PE, Brazil	22	5 T + 1 ST	5T + 1 ST	5T + 1 ST	0.79 ± 0.03	5.53
*J. multifida* L.^1^	IAC-V1P1; Instituto Agronômico (IAC), Campinas, SP, Brazil	22	4 T	4 T	2 ST	0.64 ± 0.01	5.17
*J. podagrica* Hook.^1^	LGBV-S2844; Gravatá, PE, Brazil	22	4 T, P	4 T	2 ST	0.74 ± 0.05	6.65

1 Cultivated in the green house of the Department of Genetics, Universidade Federal de Pernambuco, Recife, Brazil;

2 T – terminal signals;

3 P – pericentromeric signals in all chromosomes;

4 ST – subterminal signals.

**Figure 2:**
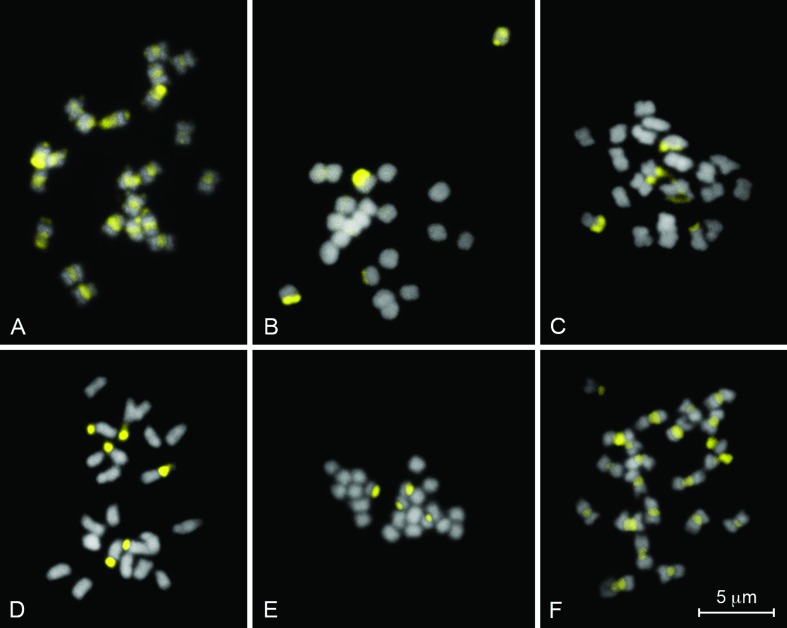
Mitotic metaphases of six *Jatropha* species stained with chromomycin A_3_ (CMA) and 4’,6-diamidino-2-phenylindole (DAPI), evidencing CMA+/DAPI^-^ bands in yellow: (A) *Jatropha curcas*; (B) *J. gossypiifolia*; (C) *J. integerrima*; (D) *J. mollissima*; (E) *J. multifida*; and (F) *J. podagrica*.

**Figure 3:**
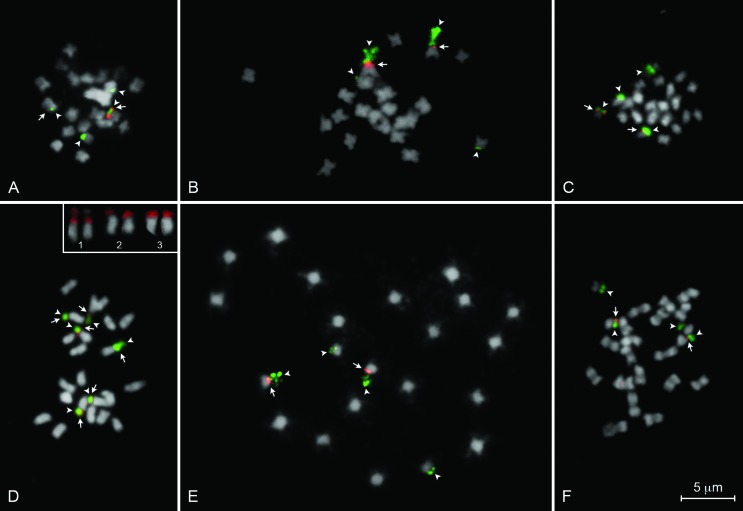
Localization of 5S (red) and 45S (green) rDNA sites in mitotic metaphases of six *Jatropha* species: (A) *Jatropha curcas*; (B) *J. gossypiifolia*; (C) *J. integerrima*; (D) *J. mollissima*; (E) *J. multifida*; and (F) *J. podagrica*. Arrows and arrowheads indicate the 5S and 45S rDNA sites, respectively. Numbers in D evidence the 5S rDNA sites in *J. mollissima*.

The CMA/DAPI staining revealed four chromosomes with terminal CMA^+^/DAPI^-^ bands for *J. curcas*, *J. gossypiifolia*, *J. integerrima*, *J. multifida* and *J. podagrica* (Figures 2A-C,E,F and 4A-C,E,F). However, *J. mollissima* diverged from the other five species by showing six terminal CMA^+^/DAPI^-^ bands (Figures 2D and 4D). In addition, *J. curcas* and *J. podagrica* presented pericentromeric CMA^+^/DAPI^-^ bands in all chromosomes, although they were not always clear, depending on the condensation level of the chromosomes (Table 1; Figures 2A,B,F and 4A,B-F). Also, depending on the chromatin condensation level, terminal CMA^+^/DAPI^-^ dots were visible in almost all chromosome arms of *J. curcas* (Figures 2A and 4A). The high amount of CMA^+^ heterochromatin (GC-rich) is in accordance to Guo *et al.* (2016), who reported an average G+C content of 65.04% for *J. curcas.* The pericentromeric heterochromatin in *J. curcas* is at least partially related to *gypsy*-type retrotransposons (Alipour *et al.*, 2014), while terminal heterochromatic dots are related to *copia*-type retrotransposons (Alipour *et al.*, 2013). These patterns of heterochromatin rich karyotypes have also been described for other Euphorbiaceae species, such as castor (*Ricinus communis* L.), *Euphorbia characias* L., *E. hirta* L., *E. hyssopifolia* L., *Manihot dichotoma* Ule and *M. esculenta* Crantz (Carvalho and Guerra, 2002; D’Emerico *et al.*, 2003; Vasconcelos *et al.*, 2010; Santana *et al.*, 2016).

**Figure 4:**
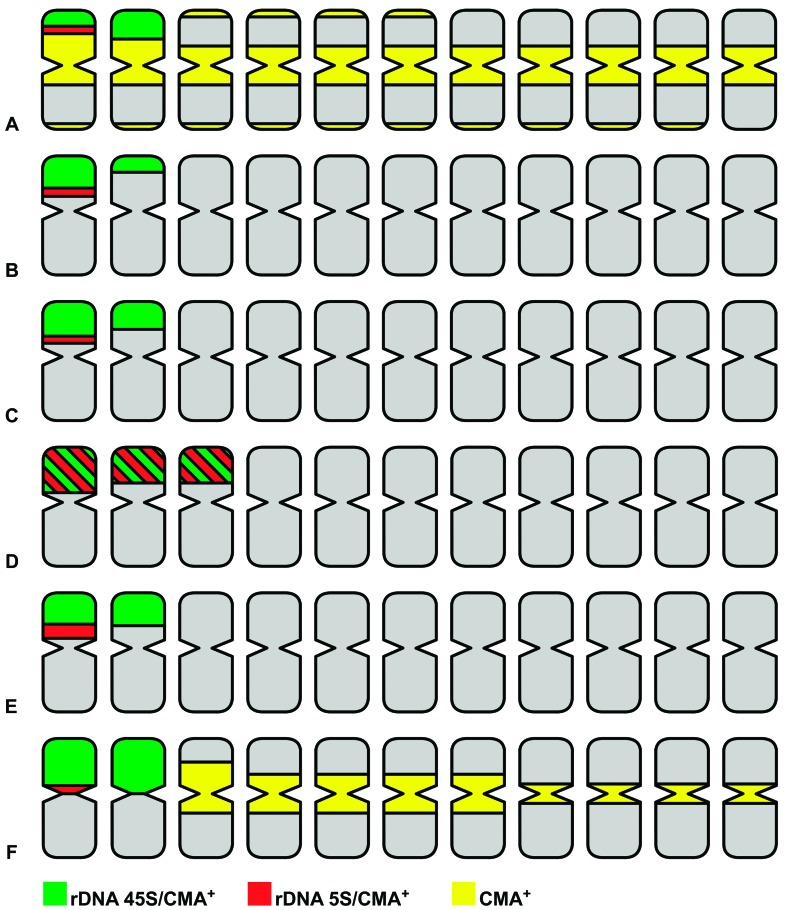
Representative idiograms of cytogenetic markers in all chromosome pairs of six *Jatropha* species. (A) *Jatropha curcas*; (B) *J. gossypiifolia*; (C) *J. integerrima*; (D) *J. mollissima*; (E) *J. multifida*; and (F) *J. podagrica*. It is important to note that only chromosome markers are evidenced in the idiograms, and actual chromosome sizes and arm ratios were not represented. Also, due to the high variation in the presence of the terminal CMA^+^ bands in the *J. curcas* karyotype, depending on the condensation level of the chromosomes, only signals always visualized were represented.

Five out of the six analyzed species (*J. curcas*, *J. gossypiifolia*, *J. integerrima*, *J. multifida*, and *J. podagrica*) presented one 5S and two 45S rDNA site pairs, both co-localized with CMA^+^/DAPI^-^ bands, with an apparent adjacency between the 5S rDNA and one of the 45S rDNA pairs (Figures 3A-C,E,F and 4A-C,E,F). On the other hand, *J. mollissima* presented six chromosomes with a co-localization between 5S and 45S rDNA sites, which also corresponded to CMA^+^/DAPI^-^ bands (Figures 3D and 4D). Furthermore, one of these three chromosome pairs of *J. mollissima* presented a heteromorphism: one of the chromosomes of the pair 1 presented a smaller 5S rDNA, not covering the satellited region (Figure 3D). Data for number and distribution of 5S and 45S rDNA sites in *J. curcas* corroborated previous data for the species (Witkowska *et al.*, 2009). For the remaining species, rDNA data are being reported for the first time in the present work.

Both the number and the distribution patterns of 5S and 45S rDNA sites seem to be quite conserved in *Jatropha*, although the superposition of 5S and 45S rDNA sites and the presence of 5S rDNA in more than one chromosome pair in *J. mollissima* are reported for the first time in a species of Euphorbiaceae (see, for instance, Carvalho and Guerra, 2002; Vasconcelos *et al.*, 2010; Santana *et al.*, 2016). Furthermore, the presence of both 5S and 45S rDNA sites in the same chromosome arm is not common in the family, and it has been observed only in *E. hyssopifolia* so far (Santana *et al.*, 2016). Nevertheless, besides the peculiar distribution pattern of the rRNA genes in *J. mollissima*, the heteromorphism observed in one chromosome pair of the species may indicate a derived karyotype within the genus. This condition may be related to active transposable elements associated with rDNA amplification, considering the abundance of such repetitive DNA in heterochromatic regions (Eickbush and Eickbush, 2007; Schubert, 2007; Roa and Guerra, 2015). For instance, the terminal regions of *J. curcas* chromosomes are rich in *copia*-type elements, including the 5S rDNA bearer (Alipour *et al.*, 2013). On the other hand, one cannot discard the possibility of additional cryptic rDNA sites that could not be evidenced by FISH in the other five analyzed karyotypes (see Cabrero and Camacho, 2008; Vasconcelos *et al.*, 2010; Roa and Guerra, 2015). The co-localization of 5S and 45S rDNA FISH sites is very uncommon in angiosperms, being reported only for a few Asteraceae species, as a consequence of an interspersed position of both unit genes (Garcia *et al.*, 2010). Such a feature hardly guarantees any evolutionary advantage due to the differences in gene functionalities between the two types of rDNAs. These are probably associated with proliferation mechanisms of transposable elements (Ciganda and Williams, 2011; Roa and Guerra, 2015), and more frequently observed in gymnosperms (Garcia and Kovarik, 2013) and in early diverging land plants (Wicke *et al.*, 2011).

The flow cytometry analysis revealed a variation of DNA content among the analyzed species ranging between 2C = 0.64 pg for *J. gossypiifolia* and *J. multifida* and 2C = 0.86 pg for *J. curcas* (Table 1). For *J. gossypiifolia* (2C = 0.64 ± 0.02 pg), *J. integerrima* (0.85 ± 0.03), *J. mollissima* (0.79 ± 0.03) and *J. multifida* (2C = 0.64 ± 0.01 pg) the genome sizes were estimated for the first time (Table 1). For *J. curcas*, the 2C-value obtained here (2C = 0.86 ± 0.02 pg) was similar to the previously reported by Carvalho *et al.* (2008) (2C = 0.85 ± 0.01 pg). On the other hand, our results for *J. podagrica* (2C = 0.74 ± 0.05 pg) were different from the obtained by Vesely *et al.* (2012) (2C = 0.60 ± 0.05 pg). This discrepancy may be occurred either due to differences between the internal reference standards used in the two analyses (Tiryaki and Tuna, 2012) or due to an intraspecific polymorphism in *J. podagrica* related to variations in the repetitive DNA content. Although the six species present the same chromosome number, the observed variation in the genome sizes (1.34x) reinforces the importance of DNA content estimations in order to understand the karyotype evolution of homoploid species (Loureiro *et al.*, 2010), helping the planning of interspecific crosses for breeding purposes.

The cytogenetic features reported here revealed different patterns of heterochromatin distribution for the first time for five *Jatropha* species and confirmed the previous data for *J. curcas*, besides allowing the identification of chromosome markers for the genus: (1) *J. curcas* and *J. podagrica* with an accumulation of pericentromeric heterochromatin; (2) five species, including *J. curcas*, with a conserved number and distribution of 5S and 45S rDNA; (3) 5S rDNA sites detected as CMA^+^; (4) *J. mollissima* as the first reported species with more than one pair of 5S rDNA sites in Euphorbiaceae and with a distinct distribution (with FISH superposition) of 5S and 45S rDNA; (5) no apparent correlation between genome size and revealed heterochromatin.

## Supplementary material

The following online material is available for this article:

Table S1 - Traits of agronomic interest of the six analyzed species of *Jatropha*.
