# Supplementary material for: Karyotype and genome size comparative analyses among six species of the oilseed-bearing genus Jatropha (Euphorbiaceae)
Source: Genet Mol Biol. 2018 May 14;41(2):442–9. doi: 10.1590/1678-4685-GMB-2017-0120 (PMC6082239; doi:10.1590/1678-4685-GMB-2017-0120)
Supplement: Supplementary file 1 [file 1415-4757-GMB-10159016784685GMB20170120-s001.pdf]

Supplementary material to "Karyotype and genome size comparative analyses among six species of the oilseed-bearing genus *Jatropha* (Euphorbiaceae)"

Table S1 - Traits of agronomic interest of the six analyzed species of *Jatropha*, as measured from cultivars and lineages produced by Instituto Agronômico (IAC; Campinas, Brazil).

Species	Average oilseed content (%)	Average O/L ratio[1]	Average phorbol content (mg/g)	Main features
J. curcas L.	11.0-39.0	1.41	0.0-8.57	High seed and oil productivity; high O/L ratio; dehiscent
J. gossypiifolia L.	47.9	0.38	3.67	High oilseed content; profuse fructification; short stature; dehiscent; drought tolerance
J. integerrima Jacq.	40.2	0.16	2.70	High oilseed content; short stature; indehiscent; resistant to diseases
J. mollissima (Pohl) Baill.	35.0	0.26	2.21	Large fruits; dehiscent; drought tolerant; resistant to pests
J. multifida L.	50.1	0.53	2.74	High oilseed content; large fruits; indehiscent; resistant to diseases
J. podagrica Hook.	42.7	0.41	3.22	Large fruits; short stature; indehiscent; resistant to fusariosis

[1]Oleic to linoleic acid ratio.
